# The Realities of Caring for a Person with a Mental Disorder in Rural and Remote Capricorn District of South Africa: A Qualitative Study

**DOI:** 10.1007/s10597-024-01360-w

**Published:** 2024-09-23

**Authors:** Thembi Nkomo, Mokoko Percy Kekana

**Affiliations:** 1 University of Limpopo, C/O R71 Tzaneen Road, Sovenga, 0727 South Africa; 2 Present Address: University of the Free State, 205 Nelson Mandela Dr, Park West, Bloemfontein, 9301 South Africa

**Keywords:** African societies, Caregiver support, Family members, Lived experiences, Mental disorder

## Abstract

In rural African communities, family caregivers shoulder the responsibility of caring for loved ones with mental disorders, often without professional support. This qualitative explorative study, conducted in Limpopo Province, South Africa, aimed to explore the realities of caring for a family member with a mental disorder in rural and remote Capricorn District, in order to uncover insights that can inform support systems, the academic community, interventions, and policies. Non-probability purposive sampling was used to ensure the reproducibility and validity of the results by focusing on participants who are actively involved in caregiving, living in the rural and remote of Capricorn District, in order to provide a comprehensive understanding of their experiences, and this resulted in 15 participants (13 females, 2 males). Data saturation determined the sample size, with data collected through in-depth interviews and analyzed using Tesch’s open-coding method. The findings revealed that cultural and spiritual beliefs strengthen caregivers, who exhibit resilience and resourcefulness, yet face financial strain, career setbacks, social isolation, and health declines. The study underscores the critical role of healthcare professionals in recognizing and addressing the challenges faced by family caregivers, while also advocating for the academic community to prioritize the development and dissemination of educational programs focused on safe and ethical coping strategies for caregivers and for policymakers to develop comprehensive mental health services that are accessible and culturally sensitive to rural and remote communities. This is essential because the well-being of caregivers directly influences the rehabilitation and community integration outcomes for individuals with mental disorders.

## Introduction

In the heart of numerous African cultures, the responsibility of caring for a family member requiring ongoing assistance, such as someone with a chronic mental disorder, has traditionally been distributed among various family members, including extended relatives (Ndlovu & Mokwena, 2023). As defined by the World Health Organisation [WHO], mental disorders encompass a spectrum of disruptions to cognitive functions, emotional regulation and behavioural patterns, often causing distress and impairment in daily life (WHO, 2022). Anyone has a threshold for developing a mental disorder, and given sufficient triggers, any person can develop a mental disorder (Weir, 2012). In the ever-changing landscape of Sub-Saharan Africa, the burden of mental disorders is on the rise and is projected to surge by a staggering 130% by 2025 (Charlson et al., 2014). Furthermore, the growth in mental disorders by 2050 is likely to significantly affect health and productivity in Sub-Saharan Africa (Charlson et al., 2014). Against this backdrop, family members shoulder the responsibility of caregiving, a role deeply rooted in cultural norms and familial obligations.

Even after persons with mental disorders have been stabilized in mental health facilities, family members commonly experience physical exhaustion, frustration, anger, fear, and other adverse emotions while providing care (Shimange et al., 2022). Although medication proved helpful, most family members usually have to plead with the patient to take it. Hospitalization of patients with mental disorders helps to give liberty and relief to the caring family members (Wankiiri et al., 2013).

Providing care for an individual with a mental disorder often entails the potential for encountering physical, psychological, and social challenges (Özgönül & Bademli, 2022). Subsequently yielding negative effects on the quality of life of the carer and the standard of care delivered (Shah et al., 2010).

Family engagement plays a vital role in the treatment and recovery of individuals with a mental disorder. Although mental health challenges often intertwine with various social and psychological dynamics within the family, the involvement of family members has been linked to relapse prevention and shorter hospital stays for individuals with mental health issues (Ong et al., 2021). Recognizing this pivotal role, the South African National Mental Health Policy Framework and Strategic Plan 2023–2030 emphasizes that social support is valued, and that maximum support should be provided to families and carers of those with mental disorders in order to broaden the network of support and care.

Yet, the reality remains that family caregivers often navigate this journey with little to no support from skilled health professionals (Wankiiri et al., 2013). This lack of support is not unique to caregivers of individuals with mental disorders; it is a challenge also faced by family caregivers of older adults with chronic diseases, as evidenced by a South African study that explored their perceptions and experiences (Mthembu et al., 2016). This parallel struggle underscores the broader systemic issues in accessing professional support for caregiving in South Africa, regardless of the nature of the care recipient’s condition. This, therefore, led to the purpose of the study, which was to explore the realities of caring for a person with a mental disorder in rural and remote Capricorn District of South Africa.

Additionally, the study directly contributes to Sustainable Development Goal (SDG)3 of ensuring healthy lives and promoting well-being for all, as well as SDG 11, which aims to make cities and human settlements inclusive, safe, resilient, and sustainable (United Nations, 2015). Furthermore, this study is pivotal in advancing knowledge within the field of mental health research by providing empirical evidence on the realities of caring for a family member with a mental disorder in rural and remote settings. This study fills a significant gap in the literature, which often overlooks the challenges faced by these populations in rural and remote contexts (Silaule et al., 2023). By shedding light on the unique realities of caring for a family member with a mental disorder in the rural and remote Capricorn District of South Africa, the study enhances understanding of the broader dynamics of mental health care in under-resourced areas. This newfound knowledge is crucial for informing policy, developing culturally sensitive interventions, and improving support systems, leading to better mental health outcomes and contributing to the global agenda of sustainable development. The study’s findings not only underscore the urgent need for targeted mental health initiatives in rural and remote communities but also demonstrate the value of research that focuses on often-neglected populations, thereby justifying the importance of this study in advancing both academic knowledge and practical applications in the field of mental health.

This study aims to explore these realities by integrating two theoretical models: the Stress Process Model (Pearlin et al., 1981) and the Double ABCX Model (McCubbin & Patterson, 1983).

The Stress Process Model (Pearlin et al., 1981) provides a foundational understanding of how stressors, such as caring for a family member with a mental disorder, affect the family caregivers’ well-being through various mediators like coping strategies and social support. This model highlights the importance of personal and contextual factors in shaping stress experiences and mental health outcomes. Studies by L’Heureux et al. (2022), Ndlovu and Mokwena (2023), and Sharif et al. (2020) highlight the unique stressors and coping mechanisms in these contexts, providing a foundation for this research. Building on this, the Double ABCX Model of Family Adjustment and Adaptation (McCubbin & Patterson, 1983) offers a family-centered perspective, elucidating how families adapt to crises. It considers the initial stressor (A), available resources (B), family perceptions (C), and the resulting crisis (X), along with subsequent adaptation processes. This model is crucial for understanding the dynamic interactions within families as they navigate the complexities of mental health care in rural settings. The theoretical models underscore the need for tailored mental health services, and this is further supported by empirical literature from the Mpumalanga Province of South Africa (Silaule et al., 2024), which underscores the challenges faced by caregivers of individuals with mental disorders in under-resourced areas.

By integrating these models, this study comprehensively examines the multi-level factors influencing the care of individuals with mental disorders in rural communities. This integrated approach not only highlights the intricate interplay between individual, family, and community-level dynamics but also underscores the critical need for targeted interventions, the academic community, and policy initiatives to support these families effectively. This then led to the research questions of what are the experiences of family caregivers when caring for a family member with a mental disorder in the rural and remote Capricorn District of South Africa? What are the challenges family members encounter while providing care and what coping skills do family caregivers employ when caring for their family member with a mental disorder?

## Methods

The study employed a qualitative exploratory research design (Creswell & Creswell, 2018) using a phenomenology approach to develop an in-depth understanding of the realities of caring for a family member with a mental disorder. This qualitative approach, informed by the Stress Process Model (Pearlin et al., 1981) and the Double ABCX Model of Family Adjustment and Adaptation (McCubbin & Patterson, 1983), allowed for an in-depth exploration of the participants’ subjective experiences, the challenges they face while providing care, and the coping strategies they employ in the line of caregiving that cannot easily be reduced to numerical values (Leedy & Ormrod, 2010). This made it suitable for the study’s aim, which is to explore the realities of caring for a person with a mental disorder in rural and remote Capricorn District of South Africa. The research questions guiding the research process and ensuring that the methodology aligned with the study objective are as follows:

1. What are the experiences of family caregivers when caring for a family member with a mental disorder in the rural and remote Capricorn District of South Africa?
2. What are the challenges family members encounter while providing care?
3. What coping skills that family caregivers employ when caring for their family member with a mental disorder? These questions guided the research process, ensuring that the methodology was aligned with the study’s objectives.

### Setting and Sample

The study was conducted at a hospital located in Capricorn District in Limpopo Province. The hospital treats and admits patients from various communities in the province. The study was conducted at the hospital’s psychiatric unit and psychiatry outpatient department. Non-probability purposive sampling method was used to ensure the reproducibility and validity of the results by focusing on participants who are actively involved in caregiving, living in the rural and remote of Capricorn District, in order to provide a comprehensive understanding of their experiences, for them to aid in answering the research questions (Kelly, 2010). The sampling strategy was guided by the Double ABCX Model (McCubbin & Patterson, 1983), ensuring the inclusion of various family dynamics of persons with a mental disorder from various rural and remote settings of the Capricorn District. Eligible family caregivers were male and female caregivers, who lived and provided care to individuals with a mental disorder and live in the remote and rural areas of the Capricorn District. Participants were purposively sampled from visitors to the psychiatric ward and outpatient department.

### Data Collection

Data were collected using in-depth semi-structured face-to-face interviews (Brinkmann & Kvale, 2018) and recorded using an audio tape recorder. A sample size of 15 participants was determined by data saturation (Saunders et al., 2018), comprising both male and female caregivers living with and caring for family members with mental disorders in the rural and remote Capricorn District. An interview guide was used to facilitate the discussion during the interview, to ensure consistency across interviews while allowing flexibility to explore emerging themes. The interview guide comprised a demographic section as well as questions to guide the interview. The leading questions were, “What are your experiences when caring for your family member living with a mental disorder?” “What are the challenges that you encounter while providing care? “What strategies do you use to cope?” The leading questions were followed by open-ended probing questions to elicit detailed information about the participants’ lived experiences, daily encounters, challenges, and coping strategies used (Polit & Hungler, 2013).

### Data Analysis

Data was analyzed using Tesch’s eight steps of data analysis method proposed by Creswell (2013), which inherently embraces an iterative process. This process began with **Step 1**, which involved listening to voice recordings, transcribing interviews, translating them from the participants’ vernacular languages (Xitsonga, Sepedi, northern Sotho) to English, and thoroughly reading the verbatim transcripts to familiarize with the data, integrating field notes into corresponding transcripts. In **Step 2**, two copies of the transcripts were made; one was the master copy, and the other was a copy to write the researcher’s thoughts. One transcript was selected for initial analysis, with underlying meanings and impressions noted in the margins, paragraphs, and pages numbered.

Throughout the analysis, an iterative approach was employed, allowing for a dynamic process where insights from later steps informed the refinement of earlier steps. **Step 3** involved analyzing each transcript, clustering related topics into columns, color-coding, and abbreviating topics into codes, the initial codes were not considered final. Instead, they were revisited and revised in light of new insights gained from subsequent steps. Similarly, in **Step 4**, where codes were written next to text segments and checked for emerging categories, the categories were not fixed but were open to change as the analysis progressed.

This iterative process continued in **Step 5**, where the most descriptive wording for topics was identified and turned into categories, with related topics grouped and interrelationships indicated. The categories were further defined and alphabetized in **Step 6**, but this was done with an understanding that the analysis was ongoing. **Step 7** involved assembling data for each category to conduct preliminary analysis and refine themes, which were then finalized in **Step 8**. However, even at this stage, themes were revisited to ensure they accurately captured the essence of the data.

This iterative approach ensured that the analysis was thorough, with each step informing and refining the next. The final themes were reported in a coherent narrative in Step 8, which included a detailed description of each theme, supported by participant quotes, and a discussion of how these findings relate to existing literature and theory. This iterative process was crucial in ensuring the depth and validity of the analysis. This included a detailed description of each theme, supported by participant quotes, and a discussion of how these findings relate to existing literature and theory.

### Trustworthiness of the Study

All the interviews were guided by the theoretical models, to explore key aspects such as stressors, resources, and coping strategies. Interview questions focused on the caregivers’ experiences, challenges, and coping strategies, aligning with the Stress Process Model (Pearlin et al., 1981) and the Double ABCX Model (McCubbin & Patterson, 1983).

The interview guide was formulated from various existing literature reviews on family caregiving for individuals with mental disorders, engaging with experts in qualitative research methods to give insight into the pertinent questions to ask to maximize the quality of the data to be collected, which were aligned with the research objectives. The interview guide was also piloted in a separate hospital, with family caregivers from the rural and remote areas, which allowed for refinement of the guide, probing, based on the feedback received from the piloted family members caring for family members with mental disorders. Data collection was further triangulated through observations to collect descriptive data on the participants’ behavior (Brink et al., 2012) and reflecting on what the participants were expressing by engaging in dialogue and repeating what they mentioned.

To enhance the trustworthiness of the research findings, an independent co-coder was employed to review and independently analyze the data provided by the researcher. The researchers further did member checking for the family caregivers to confirm the accuracy of the interpretations, ensuring that data represented their subjective experiences fully and also peer debriefing by engaging with the academic supervisor to challenge the interpretations and to ensure that the analyzed data was rigorous and balanced, this also minimized bias (Ahmed, 2024). Data yielded four themes and ten subthemes. All participants have been fully anonymized; they are identified by numbers as discussed and agreed with all participants.

While qualitative studies do not aim for statistical generalizability, the rich, detailed insights provided by the participants in this study, offer valuable understanding that can inform policy and practice in similar rural and remote settings. The diverse sample and rigorous analysis enhance the transferability of the findings to other contexts with similar characteristics.

## Results

The results are situated within various cultural contexts in the rural and remote Capricorn District of South Africa. The behaviours and experiences described by participants are influenced by personal and lived experiences, cultural norms, personal beliefs, and societal structures specific to this region.

### Demographic Information

**Table:** 

Gender	18–28 years	29–39 years	40–50 years	51–61 years	62 years and above	Employed	Unemployed
Male	2					0	2
Female	1	6	3	2	1	2	11

The study consisted of 15 family members who live and provide care to family members with mental disorders, 13 were female and 2 were male. Out of the 13 females, only two females had a job outside the caregiving responsibilities. The only two male participants were both studying at a university but living at home, traveling from home to the university on a daily basis. The majority of the family caregivers are females, unemployed, and between the ages of 29–39 years old.

### Themes and Subthemes

After analysis, the study yielded four themes and 10 subthemes, which are indicated in the table below.

**Table:** 

Themes	Subthemes
1. Family members develop both negative and positive family coping strategies to care for a mental health care user	1.1 Restrictive and shrewd measures to cope with handling a mental health care user 1.2 Supervision and positive aspects for medication-taking compliance
2. An increased level of burden on the family members caring for a mental health care user	2.1 Increasing level of financial burden 2.2 Negative impact on family members’ social life 2.3 Health effects from increased stress 2.4 Increased burden of losing valuables
3. Victims of mental health care users’ symptomatic and violent behaviour	3.1 Experiences of violent attacks and aggression from the mental health care user 3.2 Fear of the mental health care user’s abnormal behaviour and violent tendencies
4. Influences related to preferred treatment for mental illness	4.1 Family, religious and cultural influences to seek care 4.2 Perception on institutionalization of the mental health care user

#### Theme 1: Family Members Develop Coping Strategies that Enable Them to Care for a Person with a Mental Disorder

This theme is about coping strategies that were explored and are practised by family members as a result of their experiences when caring for their family member with a mental disorder at home. As a result, the theme yielded two subthemes of; Restrictive and shrewd measures to cope with handling a mental health care user and Supervision and positive aspects for medication-taking compliance, which are further explained below.

##### Subtheme 1.1: Restrictive and Shrewd Measures to Cope with Handling a Mental Health Care User

Some of the participants expressed that it becomes challenging when caring for their family member with a mental disorder at home to the point where they resort to restrictive and shrewd measures as a strategy to cope.

A sister-in-law, who takes care of her brother-in-law, who has a mental disorder, reported that:

“Eix! I ended up coming with a plan, I just crush his medication and put it in his soft porridge or relish, so he doesn’t see. But, at times, when he tastes the food and be able to taste the medication in his food, he stops eating.” (Participant 6, 35-year-old female).

A wife to a husband who has a mental disorder also shared the measures she incorporates when caring for her husband by sharing that:

“When I maybe come home and his playing music, very loud, so what I do is I take a glass of water then I put some sleeping pills inside so he can drink and all of us can be able to rest for the night. I don’t know if it’s safe, but someway somehow, it gives us peace because we are able to sleep at the end of the day.” (Participant 8, 36-year-old female).

A sister to a brother who has a mental disorder voiced her experiences:

“I remember this other time; my sister and I held him, and we beat him. I had anger towards him, and, at that point, I did not care if my unborn child was going to be hurt in the process, and we defeated him. When he realized that we conquered, he would run away, and we got used to it.” (Participant 2, 30-year-old female).

For some family members, the strategies they use come at a great cost because they are uncertain of the health implications of their methods. It, however, serves as a short-term solution, and because they get the desired results, it becomes a norm. These coping strategies reflect the Stress Process Model (Pearlin et al., 1981), which highlights how individuals adapt to stress through a combination of coping mechanisms and resources. In the context of caring for a family member with a mental disorder, these strategies—although potentially harmful—are ways for caregivers to manage the chronic stress and demands placed on them. The model explains that stressors (like the behavior of the mental health care user) lead to primary and secondary stress (emotional, physical, and financial burdens), which in turn elicit coping responses. When caregivers see immediate positive outcomes, such as the patient calming down or taking medication without resistance, they are likely to continue these practices despite potential long-term negative consequences. This cycle perpetuates a reliance on these coping strategies, underscoring the importance of addressing both the immediate needs of the caregiver and the long-term well-being of the mental health care user.

##### Subtheme 1.2: Supervision and Positive Aspects for Medication-Taking Compliance

Most participants expressed that they had to personally ensure that their family member with a mental disorder complies with medication intake as some stated that they monitor the medication intake.

A sister to a female sibling who has a mental disorder reported that:

“We monitor her medication intake every day, we always count her medication, so we know if she took it or not. When we tell her to take her medication, after taking it, the moment she sits, our mother goes to her bedroom to count the medication in order to check if she indeed took her medication.” (Participant 3, 30-year-old female).

One participant, whose only son has a mental disorder and is a university student, reported thus:

“We applied for distance learning so he can study while at home so I can supervise his medication intake.” (Participant 1, 47-year-old female).

One participant also expressed that:

“When he complies with his medication and also when he eats the food which I put his medication in, he becomes a good person, we are able to have a good conversation with him at home, all is well, and I am even able to send him to the shops.” (Participant 6, 35-year-old female).

Most caregivers have experienced the disruptiveness of the mental health care user when they do not comply with the medication intake. Hence, they try by all means to ensure that they assist their mental health care user to comply with the medication intake.

This then insinuates that family members also have to adapt to a new routine and be actively involved as they have to monitor their mental health care user’s medication intake every day and on time because, should they not monitor, the mental health care user will not comply. Certainly, family members also get a sense of purpose, satisfaction, and fulfilment when they ensure medication-taking compliance on a daily basis, as they can also see the difference between when the patient complies and when the patient does not comply.

#### Theme 2: An Increased Level of Burden on the Family Members Caring for a Mental Health Care User

This theme is about how much of a burden it is that which the family members are experiencing at home when caring for their family member with a mental disorder. This theme yielded four subthemes: Increasing level of financial burden, Negative impact on family members’ social life, Health effects from increased stress, and increased burden of losing valuables, which are further explored below.

##### Subtheme 2.1: Increasing Level of Financial Burden

Most participants experienced financial burden in the form of having to resign from work in order to take care of their mental health care user at home, whilst for some participants it is found that the mental health care user was the breadwinner and lost his or her job due to being non-functional at work, therefore increasing the level of financial burden at home.

A sister to a female sibling, who has a mental disorder, said:

“I was in Johannesburg when my mom called, which lead me to resign at work and come back home, because at home it was just my mentally ill sister and both parents, of which my parents were struggling to take care of her as the situation was bad, so I had to leave my job, resign and come back home in order to help my parents take care of my mentally ill sister”. (Participant 3, 30-year-old female).

One participant whose elder daughter has a mental disorder, expressed that:

“She was the one who was taking care of us financially, she had small jobs, doing people’s laundry and such. That’s how we survived, but now that she is sick, I also had to resign because I’m taking care of her baby. There is no one to take care of her baby while I’m working. Getting someone to help will mean I have to pay them of which I don’t have money, and her baby is 3 months old”. (Participant 14, 41-year-old female)

Most families are already struggling financially. As such, when one of the family members who is employed is suddenly diagnosed with a mental disorder, it becomes a financial tragedy and a burden for a standard family setting, adding more expenses into the household. Similarly, when a breadwinner in a family has to ensure proper and quality care of the mental health care user, leading to resigning at work—which was a source of income that exacerbated the already near-impossible situation—it also becomes a financial tragedy as compromises have to be made and in most cases, which also comes with after effects because it will then mean that a major change of lifestyle is forced into the lives of the family members.

##### Subtheme 2.2: Negative Impact on Family Member’s Social Life

When caring for the mental health care user at home, it tends to also affect family members’ social life as some tend to isolate themselves from their social life, and some have to neglect the activities in which they use to engage.

A participant whose female sibling has a mental disorder, reported:

“We moved as a family from where we were staying to a new place because people were already starting to talk about my sister, so we had to move to start a new life. Eix! It’s tough because we grew up there and it’s our home, but we had no choice but to move to a new place, new environment where they do not know my sister, so she can start a new life.” (Participant 3, 30-year-old female)

A participant whose only daughter has a mental disorder reported:

“You know I am educated neh, I never thought for a single day that any of my children can have mental illness, I started isolating myself, no longer visiting my friends, even at work I would spend most of the time alone, praying, even at work, I did not tell anyone that my daughter has mental illness I am scared of the stigma”. (Participant 11, 60-year-old female).

Caring for a family member with a mental disorder takes more than adapting to new daily routines. Some family members of mental health care users are often subjected to such because, without sound mental health, one will not have a quality life, and the fear of disclosure and stigma from community members often subjects family members to a socially debilitating environment. Consequently, society excommunicates them as they are no longer comfortable associating themselves with other people at either their workplace or at home. Unfortunately, this leads to families having to face issues that come with caring for a mental health care user in isolation. Social isolation and its effects can be understood through The Stress Process Model (Pearlin et al., 1981) where family members’ social life is compromised as they attempt to manage the stress of caregiving.

##### Subtheme 2.3: Health Effects from Increased Stress

Most participants also reported having been diagnosed with chronic illness or symptoms attributed to the experiences they face when caring for the mental health care user at home.

A wife to a husband who has a mental disorder shared that:

“You know I’ve… ever since he became that way ne, I’ve developed high blood pressure, and it’s too much to cope with both sicknesses, so it’s not that easy, and my children are also affected because I’ve noticed that they are not performing well anymore at school.” (Participant 8, 36-year-old female).

A participant, who is a mother to a son and wife to a husband who have a mental disorder, recounted that:

“I was diagnosed with high blood in 1999 when my husband was diagnosed with mental illness. It also went up now that my son was also diagnosed with mental illness.” (Participant 1, 47-year-old female).

A wife, whose husband has a mental disorder conveyed that:

“I recently went to the clinic as I was not feeling well, they checked my blood pressure and I was told that I might be having high blood pressure, but they told me to go back to the clinic for review, I do not sleep at night, I am always stressed and worried because of him.” (Participant 7, 54-year-old woman).

Based on the participants’ responses, it is without a doubt that caring for a mentally ill family member can negatively affect the carers’ health, thus compromising their health and wellbeing as well for the reason that they have to take care of their own health whilst taking care of their mental health care user at home and this resonates with a study conducted by Monyaluoe et al. (2014).

It is one thing to be diagnosed with a chronic illness due to nature and other factors, it is however devastating to be diagnosed with a chronic illness due to adverse effects of caring for a family member, as being diagnosed with chronic illness insinuates that one has to take medication on a daily basis, every day of their life, which is something that no one aspires. This is further confirmed by a study conducted in Zimbabwe by Marimbe et al. (2016).

It is human nature to aspire to have a quality life and to live a healthy life without any medication. Equally, it is devastating to have to live on medication intake and compliance as a result of caring for a family member. Family members tend to prioritize the health of their mental health care users and neglect their own health. These findings align with The Stress Process Model (Pearlin et al., 1981), where chronic stress impacts the caregivers’ health, necessitating coping strategies to manage their well-being.

#### Theme 3: Victims of Mental Health Care Users’ Symptomatic and Violent Behaviour

This theme is about is about what the family members experience when their family member with a mental disorder starts to be violent which they end up being violent. It therefore yielded two subthemes of; Experiences of violent attacks and aggression from the mental health care user and Fear of the mental health care user’s abnormal behaviour and violent tendencies, which are further explained below:

##### Subtheme 3.1: Experiences of Violent Attacks and Aggression from the Mental Health Care User

Most family members have reported having been victims of violent attacks by the mental health care users at home, whereby they reported having been physically abused, and some were even burned with a hot iron.

*A mother whose daughter has a mental disorder expressed that*:

“She also became aggressive towards me and hit me, also hitting her children with no apparent reason, she also broke my finger, and I had a fracture as a result.” (Participant 12, 71-year-old female).

A wife to a mental health care user husband also shared her experiences of being a victim of violent attacks by her husband and voice that:

“Ey! Whenever he gets sick, he starts to throw tantrums and he starts beating me up. As you can see, I have a scar here of an iron that he once used on me while it was still hot. Well, he will strangle me in front of my kids, and it wasn’t really good. So, ya he would strangle me in most cases…. Yoh! Sesi [yoh! My sister:], even in front of my neighbours.” (Participant 8, 36-year-old female).

Being violently attacked at home does not only bring physical pain, but it also leaves a permanent scar on the heart; it was also observed that when all participants shared their experiences of being a victim of a violent attack by their mentally ill family member, they became emotional. Even for those who experienced it a year ago, they also became emotional to indicate that indeed it has left a scar in their heart, which has not yet healed. Fear and vigilance in response to abnormal behavior can be understood through The Stress Process Model (Pearlin et al., 1981), where family members are constantly assessing and responding to potential threats.

##### Subtheme 3.2: Fear of the Mental Health Care Users’ Abnormal Behaviour and Violent Tendencies

Family members of patients living with a mental disorder tend to develop fear due to the mental health care users’ abnormal behaviour and violent tendencies as some family members expressed that they do not feel safe around the mental health care user and would also ensure that they do not leave their children alone with the mental health care user.

A daughter of a patient with a mental disorder shared her experiences of fearing her mother’s abnormal behaviour and violent tendencies as a mental health care user. She voiced that:

“I have to take the kids with me everywhere I go because I can’t leave them alone with my mother. Plus, one of my kids is disabled. I also don’t go to places like town when she is at home, because I can’t leave my children with her, I fear that she might relapse and hurt them.” (Participant 10, 24-year-old female).

A female participant detailed how she lives in fear at home due to her brother who has a mental disorder’s abnormal behaviour and violent tendencies at times. She shared that:

“This other night we did not sleep at all, he just woke up in the middle of the night, started swearing and breaking things in the house, when I called out to the male neighbours who usually assist me when he tends to be like that, they were not at home. So, you see my life is at risk because one day he will kill me and my children, I do not feel safe around him at times, especially when he does not take his medication.” (Participant 6, 35-year-old female).

A home is a place where one is ought to feel safe, free and at ease. However, when a family member caring for the mental health care user at home does not feel safe in the presence of the mentally ill thus being fearful and living in fear, it brings a sense of discomfort and poor quality of life as family members always have to be vigilant and ensure that children are safe at all times and are not in the same space with the mental health care user without supervision.

#### Theme 4: Influences Related to Preferred Treatment for Mental Disorders

This theme is based on the experiences of family members that have influenced their preferred treatment for their family member with a mental disorder. It yielded two subthemes of Family, religious, and cultural influences to seek care and Perception on the institutionalization of the mental health care user, which are further explained below.

##### Subtheme 4.1: Family, Religious and Cultural Influences to Seek Care

With regards to seeking care for mental health care users, most families would first consult traditional healers, and some opt for churches; however, when it gets worse, they resort to hospitals for management. Adversely for those who are married, they follow their cultural ways of doing things, such as having to inform their husbands’ family before taking major decisions concerning their husbands’ health. Some have to seek their religious help in hiding without the knowledge of their partner.

A wife to a husband who has a mental disorder, also shared her experiences on cultural influences to seek care for her mentally ill husband, she said:

“I then told his parents about his problematic behaviour, his sister came, spoke to him and also realised that my husband might be having a problem then suggested that we take him to the prophets, who gave us water, tied our waists with wools and told us that he was bewitched. From then, it became a bit better, but it became worse after a short period of time.” (Participant 7, 54-year-old female).

A sister to a female sibling who has a mental disorder, shared her experiences with regards to influences to seek care, and stated that:

“The elders in the family took her to the sangomas [traditional healer] for some days, which made it worse, then the school she is attending advised us to take her to a charismatic pastor in Pretoria. We then took her there, when she came back, she was better as she was able to write her Grade 12 final year examination, after her exams, she relapsed, they then took her back to the sangoma.” (Participant 3, 30-year-old female).

A mother to a son who has a mental disorder, also expressed her experiences when having to seek care for her son and the influences thereof, she stated that:

“I take my son to church without my husband’s knowledge because he said if I take our son to church, he will stop doing things in the house and stop helping financially. But I first wait for him to go to work, then go to church so that they can pray for my son because I am a Christian and do not believe in sangomas.[traditional healer]” (Participant 3, 47-year-old female)

It is without a doubt that human beings are spiritual beings and, in most cases, when a family member starts to show signs of a mental disorder, the first things that most families resort to is their spiritual and cultural beliefs. They will pay a lot of money for various spiritual and cultural beliefs and when there is no change, they then resort to medical interventions. However, even after being discharged from the hospital, they still return to seek for spiritual or cultural assistance even though they tried it at first and did not work out. Concurrently, a study conducted in Dar es Salaam, Tanzania by Iseselo et al. (2016) found that different consultation is sought as a way of finding solutions or treatments for their loved one. Some were advised to seek treatment from traditional healers but when they found no relief, they turned back to God and sought hospital services.

The persistence of seeking spiritual or cultural help, even after hospital discharge, underscores the cultural embeddedness of these practices. As evident in the study, mental health care in rural areas is often a blend of traditional and modern practices. Families might turn to medical interventions when traditional methods fail, but they do not entirely abandon their cultural practices. Instead, they incorporate them into the ongoing care process, creating a hybrid model of treatment that satisfies both cultural beliefs and the need for medical intervention.

##### Subtheme 4.2: Perception on Institutionalisation of the Mental Health Care User

With regards to institutionalisation of the patient, many participants were relieved when their mental health care user was admitted in hospital even though it is for short term. However, other participants stated that they would prefer their mental health care users to reside at a home where mental health care users are cared for by family members. But most of the unemployed participants preferred their breadwinner mental health care user’s to be at home rather than staying at an institution. This is done with the hope that they will get better and be stable enough to look for employment and provide for their families.

A wife to a husband, who has a mental disorder, shared her views on institutionalization of her mentally ill husband thus:

“To tell you the truth I want him at home. The problem is that when he becomes dangerous, it scares me. hence, I say he was sick for a year, and I was the only one taking care of him that whole year, until he started being aggressive. I don’t mind being with him at home, also because I am unemployed and depend on my children’s social grant, when he is better, he does piece jobs and provide for us.” (Participant 4, 29-year-old female).

A sister-in-law to a man who has a mental disorder voiced her views on institutionalization by saying:

“Yhii! I have a big problem with him staying with me, if there is such a place where mentally ill people stay and are taken care of, I would be very happy, it will also save my marriage because now my husband wants to divorce me because of his mentally ill brother as he says that I do not take care of him hence he relapses”. (Participant 6, 35-year-old female).

Taking care of a family member with a mental disorder can at times be burdensome for family members, especially when the patient relapses and does not cooperate. Furthermore, family members have to take care of their own personal needs and that of their mental health care user family member and at times it becomes challenging having to balance their personal needs and those of the patients. Hence, most family members felt that institutionalization is vital as it will enable them to have a quality life.

However, for those who are unemployed and are dependent upon the mental health care user for financial obligations, ideally, they would opt for institutionalization, but realistically, they have to endure and continue taking care of themselves at home. And, also, some mental health care users receive Disability Grant, which also serves as a source of income. Hence, should the mental health care user be institutionalized, it would mean that the Disability Grant will be contributed to the institution in order to cater to the needs of the mental health care user. As such, they, therefore, prefer the mental health care user to be hospitalized for a short stay when they relapse.

All the themes and subthemes directly address the research questions by exploring the coping strategies, burdens, and experiences of family members caring for a mentally ill relative. Each theme is linked to the theoretical frameworks, demonstrating how families navigate the complex dynamics of caregiving and highlighting areas where additional support and resources are needed. This alignment ensures that the research findings are both relevant and actionable, providing a clear roadmap for improving the well-being of both mental health care users and their caregivers.

Results from this study provides valuable insights for both academics and practitioners. For academics, it enriches the understanding of how families cope with the stress of caring for a mentally ill relative, highlighting the interplay between cultural practices, social support, and health outcomes. For practitioners, the findings underscore the need for comprehensive support systems that address both the mental health care user and their family members, advocating for interventions that reduce caregiver burden and enhance their coping strategies.

## Discussion

The study investigated the realities of caring for a person with a mental disorder in the rural and remote Capricorn District of South Africa, highlighting the profound impact on their lives and the coping strategies they employ to deal with the challenges. Among the 15 family members interviewed, 13 were female, and 2 were male, with only two of the females having a job outside their caregiving responsibilities. This gender imbalance underscores the traditional caregiving roles often assumed by women; this is consistent with Wekwete (2017), who reported that women from Sub-Saharan Africa, particularly Southern Africa, bear the brunt of care. As noted in the results, these led to significant economic and social consequences for these families because some women had to abandon their work in the cities in order to provide close supervision and care for their loved one with a mental disorder.

The analysis revealed four major themes: coping strategies, the burden of care, experiences of violence, and influences on treatment preferences. Family members develop both negative and positive coping strategies to manage the complex and often unpredictable nature of mental illness. Restrictive measures, such as covertly administering medication, are commonly used despite concerns about safety and ethical implications. For instance, one caregiver crushed medication into food to ensure compliance; this is a similar practice found in India by Janardhana et al. (2015), while another used sleeping pills to manage disruptive behavior. These tactics, although effective in the short term, highlight the desperation experienced by caregivers and align with the Stress Process Model (Pearlin et al., 1981), which explains how primary stressors and secondary stressors lead to significant life changes and coping behaviors. Despite these challenges, the Stress Process Model (Pearlin et al., 1981) also highlights instances of resilience, such as caregivers maintaining a sense of control through medication supervision. This application of the Stress Process Model (Pearlin et al., 1981) not only deepens the researchers’ understanding of the caregiving experience in a specific cultural context but also reveals the model’s utility in explaining the complex interplay of stressors, coping strategies, and outcomes among family caregivers of individuals with mental disorders. This further aligns with the Double ABCX Model of Family Adjustment and Adaptation (McCubbin & Patterson, 1983), which emphasizes how families use available resources and perceptions to manage stress and adapt to caregiving challenges. These findings are similar to those by Mphelane (2006) and reflect the dynamic interplay of stress, coping mechanisms, and adaptation within the caregivers’ experiences in the rural and remote Capricorn District.

The burden of care on family members is substantial, manifesting as financial strain, career disruption, social isolation, and health deterioration. Many caregivers had to resign from their jobs or forego employment opportunities to provide full-time care, significantly impacting household income; these findings concur with Shamsaei et al. (2015) in Iran. In cases where the mental health care user was the primary breadwinner, their illness exacerbated financial hardships, forcing families to adapt to lower living standards, reflecting the primary and secondary stressors outlined in the Stress Process Model (Pearlin et al., 1981). Socially, caregivers often withdrew from their communities to avoid stigma and judgment, further isolating them and reducing their support networks, consistent with findings by Wankiiri et al. (2013) in East Africa and Ntsayagae et al. (2019). This isolation is compounded by the stress and anxiety associated with caregiving, leading to chronic health conditions such as hypertension. The emotional toll of constant vigilance and the fear of violent outbursts from the mental health care user contribute to the caregivers’ declining health, as exemplified by several participants who developed high blood pressure due to the stress of caregiving, as also found by Monyaluoe et al. (2014) in the Free State province of South Africa. This aligns with the Double ABCX Model of Family Adjustment and Adaptation (McCubbin & Patterson, 1983), which highlights how families adapt to stressors and the impact of caregiving on family dynamics.

Experiences of violence are a significant concern, with many caregivers reporting physical abuse and aggression from mental health care users, aligning with the findings of Wankiiri et al. (2013). This not only causes physical harm but also leaves lasting emotional scars, as vividly described by participants who recounted being assaulted and living in constant fear. The unpredictable and sometimes dangerous behavior of mental health care users creates an environment of anxiety and insecurity, impacting the overall well-being of the entire household. This situation is well-explained by the Stress Process Model (Pearlin et al., 1981), which highlights the profound emotional and physical toll on caregivers dealing with chronic stressors. The Double ABCX Model of Family Adjustment and Adaptation (McCubbin & Patterson, 1983) further elucidates how families strive to adapt and cope with such severe stress, often leading to long-term negative outcomes. This study adds new insights into the specific challenges faced by caregivers in rural South Africa, emphasizing the urgent need for targeted support and interventions to address these issues.

Family members’ preferences for treatment are influenced by a complex interplay of cultural, religious, and practical considerations. Many initially seek traditional or religious interventions, only resorting to medical treatment when these methods fail, similar to findings in a study conducted in Nigeria by Jack-Ide et al. (2012). This dual approach reflects deep-rooted beliefs, and the hope that spiritual or cultural remedies might offer relief and helps to understand this preference, as it emphasizes the influence of cultural and spiritual beliefs in shaping mental health care practices in rural areas. However, the practical realities of caregiving, such as the need for financial support and the challenges of managing violent behavior, often lead families to prefer short-term hospitalization or institutionalization of the mental health care user, as per the study participants’ responses. The Double ABCX Model of Family Adjustment and Adaptation (McCubbin & Patterson, 1983) explains this shift by showing how families adapt to crises through coping mechanisms and resource management. For some, institutionalization is seen as a relief that can restore some semblance of normalcy to their lives, especially when the mental health care user’s behavior becomes unmanageable. This study provides new insights into how caregivers navigate these complex decisions, highlighting the significant role of cultural and practical factors in treatment preferences.

This study uniquely contributes to the literature by providing in-depth insights into the experiences of family caregivers in rural and remote Capricorn District, South Africa. This setting is under-researched, and the research findings shed light on the specific challenges faced by caregivers in these areas, such as the reliance on restrictive coping strategies and the significant impact on career development. The study reveals novel information about the interplay between cultural beliefs and treatment preferences, showing how caregivers navigate between traditional, religious, and medical interventions. This nuanced understanding is a valuable addition to the existing knowledge on mental health care in rural South Africa.

The study’s findings are subject to several limitations. The sample size of 15 family members may not fully represent the diverse experiences of all caregivers in the rural and remote Capricorn District. Additionally, the gender imbalance in the sample, with a majority of female participants, may skew the perspectives towards those more commonly associated with women’s caregiving roles. Furthermore, the study’s reliance on self-reported data could introduce bias, as participants’ responses may be influenced by recall or social desirability biases.

Despite these limitations, the study has significant implications for family practice, preservation, and policy. Family practice can be enhanced by providing caregivers with targeted support and resources to manage the burden of care, including financial assistance, respite care, and counselling services to address the emotional toll of caregiving. Preservation of family well-being can be achieved by promoting positive coping strategies and offering education on mental health to reduce stigma and improve treatment adherence.

Policy-wise, there is a clear need for the development of comprehensive mental health services that are accessible and culturally sensitive to rural and remote communities. This includes increasing the availability of skilled mental health professionals, integrating traditional and religious interventions into treatment plans where appropriate, and implementing support systems that acknowledge the unique challenges faced by caregivers in these settings. Additionally, policies should aim to alleviate the financial strain on families by providing subsidies or insurance coverage for mental health care, ensuring that economic barriers do not hinder access to necessary treatment.

## Conclusion

The application of the Stress Process Model (Pearlin et al., 1981) not only deepens understanding of the caregiving experience in a specific cultural context but also brings new knowledge by revealing the model’s utility in explaining the complex interplay of stressors, coping strategies, and outcomes among family caregivers in the rural and remote Capricorn District. Similarly, as noted in the study, the Double ABCX Model of Family Adjustment and Adaptation (McCubbin & Patterson, 1983) highlights how families adapt and adjust to the crises of caregiving, showcasing their resilience, resource management, and the significant influence of cultural and spiritual beliefs on caregiving practices. Integrating the models in the study, provided a nuanced understanding of the caregivers’ realities in the study, capturing the cultural, emotional, and practical dimensions of their experiences. They demonstrate how caregivers navigate the substantial burden of care, manage social isolation, health deterioration, and balance cultural and medical treatment preferences. This integrated approach not only contributes to new knowledge in rural mental health care but also informs targeted policies and practices to support family caregivers in similar settings.

The study findings highlight that, policymakers and healthcare professionals must recognize the urgent necessity of developing targeted support systems, including respite care and flexible employment opportunities, to alleviate the burden on caregivers. Community awareness initiatives are imperative to reduce stigma and foster social support networks. Moreover, the integration of cultural and religious practices into mental health services can enhance their relevance and effectiveness, bridging the gap between traditional beliefs and professional care.

The study’s emphasis on safety measures and the provision of accessible healthcare services, including mental health support, speaks to the broader implications for caregivers’ health and well-being. The findings resonate with Reinhard et al. (2008), underscoring the hidden patient phenomenon, where caregivers themselves suffer serious adverse physical and mental health consequences. This study, therefore, calls for actionable, practical, engaged scholarship, motivating the academic community to prioritize the development and dissemination of educational programs focused on safe and ethical coping strategies for caregivers. Such education is pivotal in improving the quality of life for caregivers and the well-being of those in their care.

In conclusion, this research not only expands the theoretical understanding of caregiver stress and coping in the context of mental disorders but also advocates for a paradigm shift in policy and practice. It is imperative that we move towards a more holistic and compassionate approach that recognizes the invaluable role of family caregivers and addresses their needs with the same urgency as those of the individuals with mental disorders themselves.
